# Dental caries and oral health-related quality of life in Preschoolers – introducing the Swedish version of the early childhood oral health impact scale (ECOHIS)

**DOI:** 10.1080/00016357.2023.2287235

**Published:** 2024-03-26

**Authors:** Nina Sabel, Lisa Olivia Ylander, Sandra Elizabeth Ståhlberg, Agneta Robertson

**Affiliations:** a Department of Pediatric Dentistry, Institute of Odontology, Sahlgrenska Academy, University of Gothenburg, Gothenburg, Sweden; b Folktandvården Västra Götaland, Gothenburg, Public Dental Service, Region Västra Götaland, Sweden

**Keywords:** Early childhood oral health impact scale (ECOHIS), validation studies, preschool children, health-related quality of life, dental care for children and oral health

## Abstract

**Objective:**

Experience of caries has a clearly negative impact on the quality of life in preschool children. The instrument Early Childhood Oral Health Impact Scale (ECOHIS) measures the oral health-related quality of life in preschool children (Child Impact Section) and their families (Family Impact Section). The aims of the study were to develop a Swedish version of ECOHIS and to evaluate the instrument’s reliability, validity, and internal consistency. Additionally, to analyse the oral health-related quality of life (OHRQoL) among preschool children who have experienced caries.

**Methods:**

The original ECOHIS questionnaire was translated into Swedish. Caregivers of preschool children aged 2–5 years were recruited at dental clinics in Sweden, to participate in the study and answer the Swedish version of the ECOHIS (S-ECOHIS). The internal consistency and reliability were assessed by using Cronbach’s Alpha coefficient. In order to measure the consistency of the study, the questionnaire was re-tested two weeks later for 10 of the caregivers and assessed by using intra-class correlation coefficients (ICCs). The results from S-ECOHIS were described as descriptive data and independent t-test was performed. All data were calculated using SPSS (Statistical Package for the Social Sciences).

**Results:**

S-ECOHIS was developed by translating the original English version using a double-blinded technique. A total of 274 caregivers participated in the study and completed the questionnaire. Cronbach’s Alpha was 0.84 for S-ECOHIS, 0.83 for CIS, and 0.66 for FIS. The ICC was 0.95 for the test-retest of S-ECOHIS. Among the respondents, 117 (43%) had children diagnosed with caries, while 157 (57%) had children without caries. The children with caries reported a higher total score of 5.97 (SD 6.16) of S-ECOHIS, compared to the score of the non-caries children 0.77 (SD 1.38) (*p* < 0.001).

**Conclusion:**

The Swedish version of ECOHIS that was developed demonstrates good validity, test-retest reliability, and internal consistency. The findings show that the oral health-related quality of life is adversely affected in preschool children with caries, with particular vulnerability observed among children with untreated caries. These results indicate that S-ECOHIS is suitable for use in future clinical and research endeavors.

## Background

Caries has a significant impact on the daily life of preschoolers. Concerns of oral health-related quality of life (OHRQoL) has led to the development of several instruments of self-reports and proxy-reports to measure the impact of oral health on daily life [1,2]. While most of the instruments are developed for adults, there are only a few specifically designed for children [3,4]. The Early Childhood Oral Health Impact Scale (ECOHIS) is an instrument to assess the OHRQoL in preschool children through proxy-report provided by their parents, based on the presence of caries [5]. Several studies show that children diagnosed with caries have a negatively affected quality of life, compared to children without caries [6,7]. Toothache resulting from caries not only causes pain and disrupts sleep in children [8,9], but it also affects the well-being of caregivers, leading to distress and guilt, which further contributes to a lower quality of life [10,11].

ECOHIS includes 13 questions with predetermined response options. While it has been published and evaluated in various languages, there is currently no valid Swedish version available. ECOHIS is parental answered and comprises nine questions regarding how the child is affected during daily activities (Child Impact Section, CIS), and four questions on how the family is affected by the child’s oral health (Family Impact Section, FIS) [5]. CIS encompasses four domains: Child Function, Child Psychology, Child Self-image, and Social Interaction, i.e. sleeping patterns, avoidance of certain foods, and social behaviour. The family impact section involves two domains: Parental Distress and Family Function, including aspects like loss of working hours due to dental concerns of the child. In summary, the parental answered ECOHIS examines how caries in preschool-aged children affect both the child’s and parental aspects of their lives.

In 2019, 23% of six-year-old children in Sweden were diagnosed with caries [8]. Understanding the impact of caries on society, especially when considering that nearly a quarter of preschool-aged children are affected, is still to be investigated. The ECOHIS instrument can serve as one piece of the puzzle to emphasize that caries is a more significant issue than just a dental concern.

The validation of both the original ECOHIS and its various versions has shown to be a strong and stable instrument [5,9–13]. The aim of this study was to develop a Swedish version of ECOHIS (S-ECOHIS), and to evaluate the instrument’s reliability, validity, and internal consistency. Additionally, the study aimed to analyze the OHRQoL of preschool children with experience of caries.

## Materials and methods

### Study sample

The study was designed as a cross-sectional study over a 12-month period spanning from August 2021 to August 2022. Children between the ages of 2 and 5 years visiting for re-calls, along with their caregivers, were recruited for the study from three dental clinics in the Public Dental Service of Region Västra Götaland and Region Halland. The sample was to include children diagnosed with caries, as well as children not diagnosed with caries.

The sample size calculations were based on Guedes et al. 2014, which provided the mean ± standard deviation (SD) of ECOHIS for both the caries and non-caries groups, by using https://clincalc.com/stats/samplesize.aspx [14]. According to the sample size calculations, 96 individuals were estimated to be needed in each group. Moreover, an additional 7% was included in the calculation for each group to account for any ‘don’t know’ responses and drop-outs (Ridell et al. 2015). From this, it was estimated that each group should consist of at least 102 individuals.

### ECOHIS instrument

The ECOHIS instrument was originally developed in the United States [5]. This instrument comprises 13 items divided into two sections. Each item has six different response options; and each option generates a score ranging from 0–4. The response options include *Never*, *Hardly ever*, *Occasionally*, *Often*, *Very often* and *Don’t know*. The options *Never* and *Don’t know* generate a score of 0 in the original version. The score for CIS (questions 1–9) and FIS (questions 10–13) ranges from 0–36 and 0–16, respectively.

The methodology for this article comprises three stages.

### Stage 1

#### Translation into Swedish

The original ECOHIS version was translated into Swedish by using a double-blinded technique [5]. Firstly, the questionnaire was translated from English to Swedish by a native Swedish speaker.

Secondly, the initial Swedish version was translated back into English by an independent individual who is fluent in English and Swedish, and unfamiliar with the original ECOHIS version.

The English version derived from the Swedish translation was then compared with the original ECOHIS questionnaire, and necessary adjustments were made to the final Swedish version. This final version, S-ECOHIS, was subsequently tested on the study sample to assess the clarity of the item wordings, response options, and comprehensibility of the scale, see Appendix.

### Stage 2

Prior to completing the S- ECOHIS questionnaire, caregivers were provided information regarding the aim of the study, and written consent was obtained. The caregivers answered the S-ECOHIS at a Public Dental Service visit for their child. They were instructed to consider their child’s life from birth until now, when responding to the questions.

During the dental visit, trained dentists conducted a standardized and routine clinical examination. To assess caries status, the Index of dmft (decayed, missed/extracted, filled teeth) was used and registered in the dental records [15]. Dental caries was assessed clinically. Teeth were reported as extracted if they were extracted due to caries. The Public Dental Service follows Swedish national guidelines and regularly conducts calibration for caries registration. The dmft data, registered in the dental records, was utilized for this study. Additionally, background information such as the patient’s age and gender were retrieved from the dental records.

### Stage 3

#### Data analysis

The internal consistency and reliability of the Swedish version were assessed for all sections and questions using Cronbach’s Alpha coefficient. The maximum expected value for Cronbach’s Alpha was 0.90; any value above this would indicate redundancy in the test [16]. To measure the consistency of the study, the questionnaire was re-administered to 10 caregivers two weeks later, and the intra-class correlation coefficients (ICCs) were used for assessment. The coefficient score of ICC (0) – (1), with a score minimum of 0.7, is interpreted and considered to be acceptable [16]; 1 indicates a strong relationship between the test and re-test.

Each question in ECOHIS has four options of responses, which are rated on a 4-point Likert scale (*Never* = 0, *Hardly ever* = 1, *Occasionall*y = 2, *Often* = 3, *Very often* = 4). The answer *Don’t know* in this study is calculated as the mean from the other responses in the same domain.

The dichotomized item responses *Occasionally, Often* and *Very often* were considered indicative of having had the experience, while the responses *Never* and *Hardly ever* were considered indicative of not being acquainted with the item. The results from the ECOHIS questionnaire were analyzed using independent t-test to compare the mean total scores between the caries and non-caries groups. Additionally, independent t-tests were conducted for the domains within the Children Impact Section (CIS) and the Family Impact Section (FIS). Comparisons between the groups concerning proportions were conducted by using Chi-2. A p-value of <0.05 was statistically significant.

All data were calculated using SPSS (25.0) (Statistical Package for the Social Sciences).

#### Ethical considerations

Ethical approval was obtained from the Swedish Ethical Review Authority (Dnr 2021-04880, September 2021).

## Results

### Study sample

Out of the initially selected children, all 274 caregivers chose to participate in the study. Among them, there were 145 (53%) boys and 129 (47%) girls. The mean age was 4.0 years for all children, with boys having an average age of 4.0 years and girls having an average age of 3.9 years. The age of the children ranged between 2 and 5 years old. The mean dmft score was 2.0 (std 3.235) ranging between 0–16.

### Caries group

Among the children, 117 (43%) had experienced caries (dmft > 0) and are referred to as the *caries group*. This group comprised of 64 boys and 53 girls, with a mean age of 4.2 years. The mean dmft score was 4.7 (std 3.477) ranging from 1–16.

Furthermore, two subgroups in the caries group were established: Children treated for caries (dmft > 0 where *d* = 0) and children who had not yet undergone treatment (*d* > 0). Analyses with independent t-test were conducted comparing the differences between these subgroups.

### Non-caries group

The *non-caries group* consisted of 157 (57%) children and had a score of dmft = 0, including 81 boys and 76 girls, with a mean age of 3.8 years.

### Stage 1

All items were translated from English to Swedish, resulting in the S-ECOHIS (Appendix 1)

### Stage 2

The S-ECOHIS item statistics and distribution of responses are presented in Tables 1 and 2. No missing responses were found. Caregivers reported that *How often has your child pain from mouth, jaws or teeth because of dental problems or treatments* was the most commonly experienced item in the CIS section of the S-ECOHIS (11%).

**Table 1:** Descriptive data of S-ECOHIS.

Impact sections and domains	Number of items	Range potential	Range results	Mean (SD) results
CIS				
Child symptoms	1	0–4	0–4	0.46 (0.81)
Child function	4	0–16	0–16	0.82 (1.82)
Child psychology	2	0–8	0–8	0.37 (1.01)
Child social	2	0–8	0–5	0.12 (0.56)
Total	9	0–36	0–33	1.77 (3.42)
FIS				
Parental distress	2	0–8	0–8	0.86 (1.68)
Family function	2	0–8	0–5	0.37 (0.85)
Total	4	0–16	0–10	1.22 (2.17)
Total S-ECOHIS	13	0–52	0–42	2.99 (4.89)

CIS: Child Impact Section, Child Social: Child self-image and social interaction, FIS: Family Impact Section, SD: standard deviation.

**Table 2:** Data obtained from 274 caregivers regarding their responses to the 13 items in S-ECOHIS.

Items of ECOHIS	Mean (SD)	‘Never’ or ‘Hardly ever’ N (%)	‘Occasionally’, ‘Often’ or ‘Very often’ N (%)	‘Don’t know’ N (%)
1. Pain	0.46 (0.81)	243 (87.6)	31 (11.3)	3 (1.1)
2. Drinking	0.22 (0.61)	253 (92.3)	15 (5.5)	6 (2.2)
3. Eating	0.27 (0.27)	249 (90.8)	20 (7.2)	5 (1.8)
4. Pronouncing	0.14 (0.61)	253 (92.3)	9 (3.3)	12 (4.4)
5. Absence	0.21 (0.61)	256 (93.4)	16 (5.9)	2 (0.7)
6. Sleeping	0.19 (0.61)	254 (92.7)	15 (5.5)	5 (1.8)
7. Irritation	0.18 (0.54)	250 (91.2)	15 (5.5)	9 (3.3)
8. Smiling	0.08 (0.38)	262 (95.6)	8 (2.9)	4 (1.5)
9. Talking	0.04 (0.26)	264 (96.3)	4 (1.4)	6 (2.2)
10. Upset	0.31 (0.80)	243 (88.7)	28 (10.2)	3 (1.1)
11. Guilty	0.55 (1.08)	221 (80.6)	50 (18.2)	3 (1.1)
12. Work	0.30 (0.68)	246 (89.8)	25 (9.1)	3 (1.1)
13. Financial	0.07 (0.35)	264 (96.3)	6 (2.2)	4 (1.5)

The mean and standard deviation are listed, as well as number and proportion (%) of no experience (Never and Hardly ever) and experience (Occasionally, Often and Very often). Number and proportion of Don’t know is shown separately. SD: standard deviation, N: number.

The response rate for Don’t know varied from 0 to 7% across the 13 items, with *How often has your child difficulties pronouncing any words because of dental problems or treatments* being the most frequently reported item with Don’t know.

### Stage 3

The internal consistency reliability was assessed using Cronbach’s alpha resulting in 0.83 and 0.66, respectively for the child and family sections. For the total S-ECOHIS, including all items, the Cronbach’s alpha internal consistency reliability was 0.84.

The test-retest showed no difference in mean score of total S-ECOHIS, CIS, FIS, or any domain, when analyzed with independent t-test. The test-retest reliability was calculated *via* ICC (intraclass correlation coefficient), showing to be 0.95 of the total score of S-ECOHIS. The ICC for CIS was calculated to be 0.99, and for FIS, it was calculated to be 0.96.

For all participants the scores of CIS ranged from 0-33, with a mean of 1.77 (SD = 3.42). The highest domain within CIS was *Child function*, with a mean of 0.82 (SD = 1.82). In FIS, the scores ranged from 0-10, with a mean of 1.22 (SD = 2.17), see Table 1. The highest domain within FIS was *Parental distress*, with a mean of 0.86 (SD = 1.68). The distribution of responses to the items are listed in Table 2.

All item responses of *Occasionally, Often* and *Very often* were reported to a larger extent in the caries group, compared to the non-caries group (Chi-2, *p* < 0.05). In CIS, the item *How often has your child pain from mouth, jaws or teeth* was reported in 11% of all respondents, with a larger proportion (23%) in the caries group, compared to the non-caries group (Chi-2, *p* < 0.001). In FIS, the item *How often have you or another family member felt guilty because of your child’s dental problems or treatment* was reported in 18% of all caregivers, with a higher proportion in the caries group (40%), compared to 2% in the non-caries group (Chi-2, *p* < 0.001).

**Figure 1:**
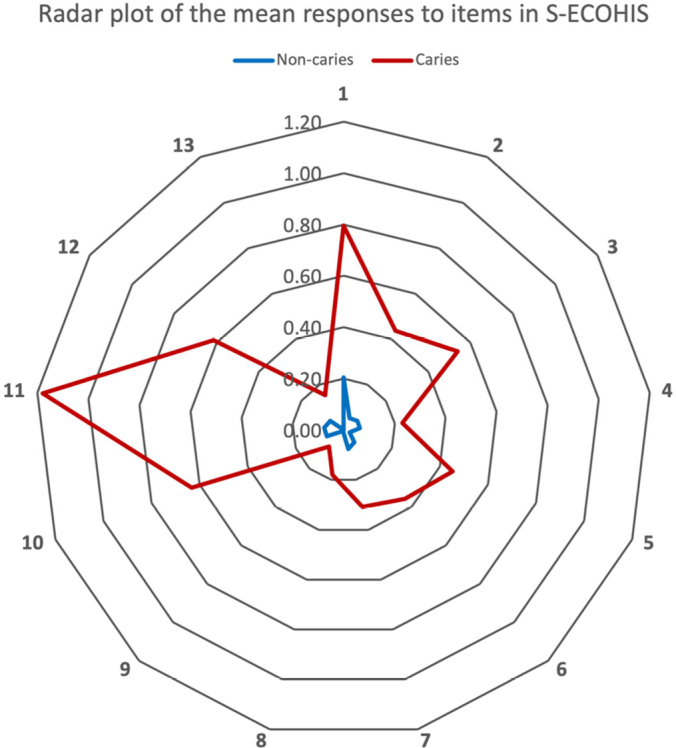
Radar Plot of the mean responses to items in S-ECOHIS.

Furthermore, the mean scores of all items were higher in the caries group, compared to the non-caries group (independent t-test *p* < 0.05), see Figure 1.

The analysis of the mean scores of S-ECOHIS between the caries group and the non-caries group is presented in Table 3(a). All domains showed significant differences between the groups, with caregivers in the caries group scoring higher, compared to the non-caries group (*p* < 0.005).

**Table 3a:** Statistical data for S-ECOHIS in two groups: The caries group (N = 117 children) and the non-caries group (N = 157 children).

Section	Domain	CARIES		NON-CARIES		*p*
		Mean	SD	Mean	SD	
S-CIS	Child Symptoms	0.80	1.02	0.20	0.46	<0.001
	Child Function	1.67	2.47	0.20	0.61	<0.001
	Child Psychology	0.67	1.35	0.14	0.57	<0.001
	Child Social	0.27	0.83	0.01	0.11	<0.005
	Total	3.38	4.63	0.56	1.05	<0.001
S-FIS			Parental Distress	1.81	2.06	0.15	0.78	<0.001
Family Function	0.77	1.15	0.06	0.24	<0.001
Total	2.58	2.63	0.21	0.83	<0.001
S-ECOHIS	Total	5.97	6.16	0.77	1.38	<0.001

The p is the p-value calculated using independent samples t-test of equality of means of the groups, equal variances not assumed (Levene’s test for equality of variances). Child Social: Child self-image and social interaction, SD: Standard deviation.

Furthermore, the two subgroups in the caries group i.e. children being treated for caries and children with untreated caries, were further analysed. Among children in the caries group, 15% (*n* = 18) had undergone dental treatment (dmft > 0 where *d* = 0), while 85% (*n* = 99) had untreated teeth (*d* > 0).

Independent t-test was performed to compare the mean of the score of the responses. Caregivers of children who had received dental treatment reported lower total scores in S-ECOHIS, compared caregivers of untreated children, see Table 3(b). Similar patterns were observed for the scores of the child and family sections.

**Table 3b:** Statistical data for S-ECOHIS of the subgroups, treated (N :18) and untreated (N: 99) children, within the caries group.

Section	Domain	TREATED (N: 18)		UNTREATED (N: 99)		*p*
		Mean	SD	Mean	SD	
S-CIS	Child Symptoms	0.33	0.59	0.88	1.06	<0.005
	Child Function	0.72	1.23	1.82	2.60	<0.05^a^
	Child Psychology	0.17	0.38	0.76	1.44	<0.001
	Child Social	0.17	0.51	0.28	0.88	ns^a^
	Total	1.34	1.97	3.75	4.89	<0.001
S-FIS	Parental Distress	0.78	1.35	2.00	2.11	<0.005
	Family Function	0.33	0.60	0.85	1.21	<0.005
	Total	1.11	1.52	2.85	2.71	<0.001
S-ECOHIS	Total	2.50	3.02	6.60	6.39	<0.001

The p is the p-value calculated using independent samples t-test of equality of means of the groups, equal variances not assumed (Levene’s test for equality of variances).

a indicating equal variances assumed (Levene’s test for equality of variances). Child Social: Child self-image and social interaction, SD: Standard deviation, ns: non-significant.

## Discussion

Caries is a continuous global problem and should not be overlooked. Having an instrument capturing children’s point of view of their dental experience is crucial. Therefore, there is a clear need for a Swedish version of the ECOHIS questionnaire, to assess the impact of dental care and oral health in preschool children in Sweden. In conformity with articles in different languages of the instrument, the Swedish translation of ECOHIS was based on the English version [9–13,17,18]. There were no difficulties concerning the translation process to Swedish, considering the highly comprehensive results showing few responses of *Don’t know* in the S-ECOHIS version. The response option *Don’t know* is of significance as a confirmation that the caregiver considered and understood the questions in the questionnaire. In the S-ECOHIS, a *Don’t know* response for a single item is recalculated as the mean of the items in the domain. In contrast, the French, German, and original version discriminate, as *Don’t know* is given a score of zero [5,11,13]. Consequently, a *Don’t know* response that influences the score of ECOHIS will give rise to a negative impact of the caries experience, rather than a positive impact or non-impact.

A score of Cronbach’s Alpha >0.7 is considered acceptable for measuring internal consistency [16]. The result for the S-ECOHIS (α = 0.84) is satisfactory and indicates good reliability. The Cronbach’s Alpha of S-ECOHIS is congruent with the Lithuanian version (α = 0.87) and French version (α = 0.82) [13,17]. The test-retest reliability of S-ECOHIS was calculated *via* ICC for the total score and its two sections, CIS and FIS, which indicates excellent agreement between the test and retest groups. The total ICC score of S-ECOHIS aligns with the findings of the French version (0.95) [13]. In comparison, the German version and the Chinese version yielded total scores of 0.81 and 0.61, respectively [10,11]. The original ECOHIS version by Pahel et al. showed a ICC score of 0.84 [5].

The results highlight the impact of caries on the oral health-related quality of life (OHRQoL) of both children and their families, as observed in previous studies conducted in other countries [5,11]. Families are affected with parents often reporting feelings of guilt when their child is diagnosed with and affected by caries, a finding consistent with prior research [8]. These findings should be taken into consideration when meeting children and parents in clinical settings. Studying OHRQoL with input from children and analysing those with or without the experience of caries, provides valuable insight regarding the child’s situation, underscoring the importance of planning and organizing dental care for preschoolers. Children are profoundly affected by caries, particularly when it remains untreated. The substantial results obtained from S-ECOHIS can inform and shape attitudes toward future dental care. Previous studies have demonstrated that treating caries in preschool children can improve their OHRQoL [19,20]. Furthermore, research suggests that ECOHIS can assist in prioritizing dental care for preschoolers with caries, including treatment under general sedation [21]. The results of S-ECOHIS show that multi-focused dental care for children, including prevention as well as treating caries in the deciduous dentition, is of importance when considering the quality of life of young children.

The sample size could be considered a limitation. However, this study was performed at three clinics with children from families with varying socio-economic backgrounds and cultures, which should be viewed as a positive aspect. Children in socio-economic disadvantaged areas tend to have a higher rate of caries, compared to the general population [22,23]. Children living in socio-economically deprived areas are not only vulnerable to life’s challenges, but also face the additional risk of experiencing a lower quality of life due to the impact of caries. Additionally, children diagnosed with caries are at an increased risk of developing caries during their adolescence [24]. Prevention in dental care is essential for all children, not just for developing caries but for health-promoting reasons in general. Moreover, the significance of a Swedish version of ECOHIS is relevant in a country where dental care is free of charge for individuals up to the age of 19.

In summary, the findings of this study confirm that care-givers of children with caries report a higher S-ECOHIS score, indicating a negative impact of caries on oral health-related quality of life in preschool children. Studying how caries in preschool children affects the quality of life, both for children and their families, is crucial for current and future dental care. The results from the S- ECOHIS demonstrate good validity, test re-test reliability and internal consistency, similar to other available translations [9–13,17,18]. These results affirm the suitability of S-ECOHIS for use in both clinical and research works.

## Conclusion

The Swedish version of ECOHIS that was developed demonstrates good validity, test-retest reliability, and internal consistency. The findings show that the oral health-related quality of life is adversely affected in preschool children with caries, with particular vulnerability observed among children with untreated caries. These results indicate that S-ECOHIS is suitable for use in future clinical and research endeavors.
